# *Birth in Brazil II*: a postpartum maternal, paternal and child health research protocol

**DOI:** 10.1590/0102-311XEN249622

**Published:** 2024-04-29

**Authors:** Mariza Miranda Theme, Márcia Leonardi Baldisserotto, Tatiana Henriques Leite, Marilia Arndt Mesenburg, Ana Claudia Santos Amaral Fraga, Maria Pappaterra Bastos, Rosa Maria Soares Madeira Domingues, Silvana Granado Nogueira da Gama, Sônia Azevedo Bittencourt, Marcos Nakamura-Pereira, Ana Paula Esteves-Pereira, Maria do Carmo Leal

**Affiliations:** 1 Escola Nacional de Saúde Pública Sergio Arouca, Fundação Oswaldo Cruz, Rio de Janeiro, Brasil.; 2 Universidade do Estado do Rio de Janeiro, Rio de Janeiro, Brasil.; 3 Universidade Federal de Pelotas, Pelotas, Brasil.; 4 Instituto Nacional de Infectologia Evandro Chagas, Fundação Oswaldo Cruz, Rio de Janeiro, Brasil.; 5 Instituto Nacional de Saúde da Mulher, da Criança e do Adolescente Fernandes Figueira, Fundação Oswaldo Cruz, Rio de Janeiro, Brasil.

**Keywords:** Pospartum Depression, Post-Traumatic Stress Disorders, Mental Health, Postnatal Care, Depressão Pós-parto, Transtornos de Estresse Pós-traumático, Saúde Mental, Cuidado Pós-Natal, Deprésion Posparto, Trastornos por Estrés Postraumático, Salud Mental, Atención Posnatal

## Abstract

Pregnancy, parturition and birth bring major changes to the lives of mothers and fathers. This article presents a research protocol for estimating the prevalence of postpartum mental health outcomes in mothers and fathers, abuse and satisfaction in delivery/abortion care, and the correlations between them and socioeconomic, obstetric, and child health factors. As a 2-component research, it consists of a prospective cohort study with all postpartum women interviewed in the 465 maternity hospitals included at the *Birth in Brazil II* baseline survey conducted from 2021 to 2023, and a cross-sectional study with the newborns’ fathers/partners. Interviews will be conducted via telephone or self-completion link sent by WhatsApp with the mother at 2 and 4 months after delivery/abortion. Partners will be approached three months after birth (excluding abortions, stillbirths and newborn death) using the telephone number informed by the mother at the maternity ward. Postpartum women will be inquired about symptoms of depression, anxiety and post-traumatic stress disorder, abuse during maternity care and quality of the mother-newborn bond. Maternal and neonatal morbidity, use of postnatal services, and satisfaction with maternity care are also investigated. Fathers will be asked to report on symptoms of depression and anxiety, and the quality of the relationship with the partner and the newborn. The information collected in this research stage may help to plan and improve care aimed at the postpartum health of the mother-father-child triad.

## Resumo

A gravidez, o parto e o nascimento são momentos de grandes mudanças na vida das mães e dos pais. Este artigo tem como objetivo apresentar o protocolo da pesquisa para estimar a prevalência dos desfechos em saúde mental nas mães e pais no pós-parto, dos maus tratos e satisfação na atenção ao parto/abortamento, e as inter-relações entre eles e fatores socioeconômicos, obstétricos e da saúde da criança. A pesquisa tem dois componentes: estudo de coorte prospectiva com todas as puérperas entrevistadas nas 465 maternidades incluídas na linha de base da pesquisa *Nascer no Brasil II* realizada entre 2021 e 2023, e estudo seccional com os companheiros/pais dos bebês. As entrevistas são realizadas por ligação telefônica ou *link* de autopreenchimento enviado por WhatsApp com as puérperas aos 2 e 4 meses após o parto/aborto. Os companheiros são abordados três meses após o nascimento (excluídos os abortos, natimortos e neomortos), a partir do telefone informado pela puérpera na maternidade. As entrevistas abordam, entre as puérperas, sintomas de depressão, ansiedade e transtorno de estresse pós-traumático, maus-tratos na atenção na maternidade e qualidade do vínculo mãe-bebê. São investigados também a presença de morbidade materna e neonatal, utilização de serviços pós-natais, e satisfação com o atendimento na maternidade. Entre os pais, é abordada a ocorrência de sintomas de depressão e ansiedade, e a qualidade do relacionamento com a esposa/companheira e o bebê. As informações coletadas nessa etapa da pesquisa poderão subsidiar o planejamento e melhoria do cuidado voltado para a saúde da tríade mãe-pai-filho após o nascimento.

## Resumen

El embarazo, el parto y el nacimiento son momentos de grandes cambios en la vida de madres y padres. Este artículo tiene como objetivo presentar el protocolo de investigación para estimar la prevalencia de los resultados de la salud mental en madres y padres en el posparto, maltratos y la satisfacción durante la atención del parto/aborto, y las interrelaciones entre ellos y los factores socioeconómicos, obstétricos y de salud infantil. La investigación tiene dos componentes: un estudio de cohorte prospectivo con todas las puérperas entrevistadas en las 465 maternidades incluidas en la línea de base de la encuesta *Nacer en Brasil II* realizada entre 2021 y 2023, y un estudio seccional con las parejas/padres de los bebés. Las entrevistas se efectúan mediante llamada telefónica o enlace de autocumplimentación enviado vía WhatsApp a las puérperas a los 2 y 4 meses después del parto/aborto. El contacto con la pareja se hace a los tres meses del nacimiento (excluyendo abortos, mortinatos y muertes de recién nacidos), a través del teléfono facilitado por la puérpera en la sala de maternidad. Las entrevistas abordan, entre las puérperas, los síntomas de depresión, ansiedad y trastorno de estrés postraumático, maltrato durante la atención en la maternidad y la calidad del vínculo madre-bebé. También se investiga la presencia de morbilidad materna y neonatal, uso de servicios posnatales y satisfacción con la atención en la maternidad. Entre los padres, se aborda la ocurrencia de síntomas de depresión y ansiedad, y la calidad de la relación con la esposa/pareja y el bebé. La información recopilada en esta etapa de la investigación puede apoyar la planificación y mejora de la atención dirigida a la salud de la tríada madre-padre-hijo después del nacimiento.

## Introduction 

Pregnancy, parturition and birth bring major changes to the lives of mothers and fathers. During postpartum, in particular, they are preoccupied with the newborn’s demands and care, having to adjust to the new reality ^1^. The first few weeks postpartum are an important and special period. Health services can play an essential role in this period, promoting family care by encouraging good practices and detecting problems that affect newborn health and the parents’ physical and emotional health.

The World Health Organization (WHO) states that postnatal care efforts should go beyond increasing coverage and include quality of care as a core issue, ensuring the provision of care for women, newborns, partners, parents, caregivers and families while respecting the cultural context. Which includes providing effective clinical practices, relevant and timely information, as well as psychosocial and emotional support ^2^.

Complying with the WHO recommendations, the Brazilian Ministry of Health recommends qualified and humanized postnatal care, with actions that integrate promotion, prevention and health care. The recommendations include assessing the health status of the mother and the newborn; providing breastfeeding support; identifying risk situations or complications; evaluating the mother-newborn interactions; assessing maternal psycho-emotional conditions; and offering guidance on reproductive planning ^3^. 

However, studies on the quality of postnatal care in Brazil reveal, in addition to the low coverage of consultations up to 42nd postpartum day (from 16.8% to 58%), that the health professionals mostly focus on breastfeeding encouragement and guidance regarding contraception ^4^. Important actions related to the mothers’ physical and emotional health are infrequent. This picture is even more worrisome among adolescents: less than half of postpartum consultations were classified as adequate ^4^. 

Analyzing data from the *Birth in Brazil I* survey conducted between 2011/2012, Domingues et al. ^5^ observed that attendance to birth review visits in the first 15 days postpartum and to routine newborn visits in the first week of life were unsatisfactory, reaching values lower than 50%. In this same research, joint analysis of the indicators of effective use of post-natal services in public health system units showed that only 1.5% of mothers and their babies received all the recommended care ^6^. 

Postpartum is a delicate period and requires close attention to the risks of maternal morbidity and mortality, identifying serious complications such as bleeding, infection, hypertensive disorders and diabetes mellitus, as well as common complications such as breastfeeding difficulties ^7^. Among neonates, the highest infant mortality rate is observed in the first 28 days of life, and its reduction is related to early identification of severe morbidities and their primary causes ^8^. Moreover, this is an important period for carrying out preventive practices, such as vaccination and neonatal screening tests. 

Given this scenario, identifying the obstacles that may be contributing to underutilization of these services is paramount. Besides the socioeconomic barriers imposed in Brazil, an important obstacle to the use of postnatal health care services are the reports of abuse during delivery/abortion care. Research estimates that 44% of women experience at least one act of physical abuse, psychological abuse, disrespectful treatment, lack of privacy, difficulty asking questions about their health status or that of their newborn, and loss of autonomy. Experiences of abuse contribute to the breakdown of trust in health services, decreasing the likelihood that a woman and her newborn will use these services after delivery ^9^. Moreover, childbirth care mistreatment can affect women’s mental health, increasing the risk of developing postpartum depression ^10^ and post-traumatic stress disorder (PTSD) ^11^.

Postpartum depression is the most common psychological condition after birth, with a global average prevalence of 17% among women with no previous history of depression ^12^. In Brazil, postpartum depressive symptoms affect more than 25% of women ^13^. Moreover, growing evidence shows that postpartum women are also at risk of developing PTSD due to stressful experiences related to parturition ^14^. Research estimates that 25% of women experience PTSD symptoms after giving birth to a healthy full-term baby ^15^. Co-occurrence of depression and PTSD seems to be caused by traumatic childbirth experience ^16^.

Importantly, studies that evaluated postpartum follow-up in Brazil make no reference of actions concerning fatherhood. Becoming a father gives a man a new identity that comes with changes in his priorities. Fatherhood in the perinatal period can be complex and require new responsibilities. Studies point to a potential increase in stressors, with a higher prevalence of emotional disorders such as depression and anxiety ^17^ ^,^ ^18^. 

Moreover, the co-occurrence of depression in both parents during postpartum has consequences on a larger scale, because the well-being and mental health of mother, father and baby are closely related. Parental perinatal complications can interfere with the parent-child relations, bonding with the newborn, and the child’s emotional development, with an increased risk of aggressive behavior, attention problems, and externalizing and internalizing behaviors in the first years after birth ^19^.

Thus, postnatal follow-up should approach the family in a comprehensive and effective manner, detecting early on situations that affect their well-being ^2^ ^,^ ^3^. Given the complexity of postpartum for mothers and fathers, this article presents the follow-up protocol of interviews with postpartum mothers participating in the first stage of the *Birth in Brazil II: National Research on Abortion, Labor and Childbirth* (*Birth in Brazil II*), and their partners, and to estimate the prevalence of maternal and paternal postpartum mental disorders, of mistreatment and satisfaction with delivery/abortion care, and the correlations between them and socioeconomic, obstetric, and child health factors. The information collected in this research stage may help to plan and improve care aimed at the postpartum health of the mother-father-child triad. 

## Method

### Baseline of *Birth in Brazil II*

The *Birth in Brazil II* survey uses a nationally and regionally representative sample of hospital births in Brazil, with data collected from 2021 to 2023, including 22,050 postpartum women and approximately 2,205 women hospitalized for abortion care and 22,000 for childbirth care. The 2-stage probabilistic sampling process, corresponding to maternity and postpartum women, included 465 maternity hospitals stratified according to the country’s macroregion (North, Northeast, Southeast, South, Central-West); type of hospital (public, mixed or private); location (capital and municipalities of the metropolitan region/other cities), and number of live births/year (100-499 live births/year, ≥ 500 live births/year). Maternity hospitals with 100-499 live births/year were selected systematically after classification by stratum and number of live births. Maternity hospitals with 500 and more live births/year were selected by probability proportional to size according to the number of live births. We defined a fixed sample of 30 interviews for maternity hospitals with 100 to 499 live births/year and of 50 interviews for maternity hospitals with 500 and more live births/year. Postpartum women were included sequentially, according to date and time of delivery, until the sample for each maternity hospital was reached. Maternity hospitals with less than 100 live births/year contribute with 2% of all births in the country, and their exclusion aimed to obtain the best cost-effectiveness ratio without compromising the quality of the estimates.

Besides collecting data from medical records, prenatal cards and ultrasound results, we interview postpartum women at the maternity ward six hours after birth/abortion. Each interview lasts an average of 40 minutes and addresses various socioeconomic and behavioral aspects, prenatal care, admission to the maternity ward, parturition and immediate postpartum, in addition to the abortion circumstances and procedures performed. We planned two follow-up interviews with the women after delivery/abortion and one interview with the babies’ fathers after birth. All questionnaires were developed specifically for the research and the collected data entered into the REDCap platform (https://www.redcap.fiocruz.br/redcap/). Interviews are conducted using tablets and the answers are electronically recorded and stored on the Oswaldo Cruz Foundation (Fiocruz) server. Further details about the *Birth in Brazil II* survey are published in Leal et al. ^20^. 

### A postpartum maternal, paternal and child health study

This study includes two components: a prospective cohort with all postpartum women interviewed at the *Birty in Brazil II* baseline at 2 and 4 months after delivery/abortion and a cross-sectional study with the babies’ fathers three months after birth. The first follow-up interview with postpartum women seeks to investigate the prevalence of maternal and neonatal morbidity, postnatal health service use, satisfaction with abortion care, childbirth-related symptoms of depression, anxiety, and PTSD, and mother-child bonding. In the second follow-up, we investigate the occurrence of abuse during delivery/abortion care, satisfaction with parturition care, everyday discrimination, and breastfeeding at four months. As for the fathers, we intend to estimate the prevalence of depression and anxiety, and the quality of the relationship with the partner and the newborn. Moreover, we will test the correlations between several maternal, neonatal, and paternal outcomes and socioeconomic variables, pregnancy planning, prenatal care, obstetric history, and labor and birth care, obtained during the maternity interview, and the infant’s health conditions after discharge. 

### Inclusion criteria

All women interviewed at the maternity ward, who authorized subsequent contact, are eligible to participate in the follow-up at 2 and 4 months after delivery/abortion. Fathers who had the contact phone number provided by the partner and whose babies are alive at the time of the interview (excluding miscarriages, stillbirths, and newborn deaths) are eligible for the interview.

### Exclusion criteria

A man who reports that he is no longer the woman’s partner at the time of the interview is considered ineligible. 

### Contact strategies 

**Figure 1:**
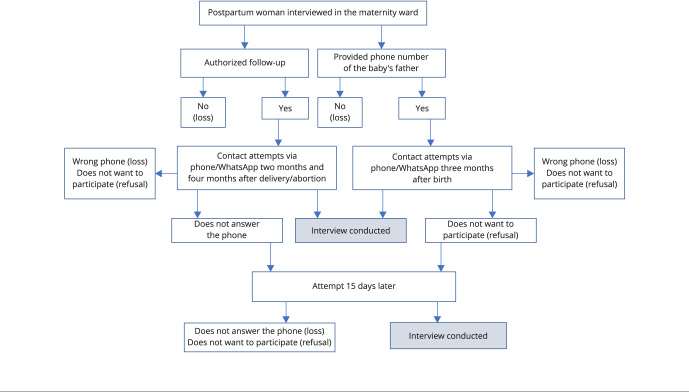
Flowchart of the interviews with the postpartum women and the babies’ fathers. *Birth in Brazil II* survey, 2021-2023.

Upon admission to the maternity ward, women provide various forms for contact, including home and mobile phone (hers, her partner’s, and other relatives’), email, and social media (Instagram and Facebook), to broaden the possibility of future contacts. Invitation to participate is made by phone or message application, on different days and times, from 9:00 a.m. to 8:00 p.m., including weekends. The study planned at least five contact attempts. The first follow-up interview lasts an average of 25 minutes, the second follow-up 15 minutes, and the interview with the father 15 minutes. They are done by phone, which can be scheduled, or by a self-completion link sent by WhatsApp, if the phone interview is not possible due to time unavailability or other reasons. If the interview is interrupted for any reason, a new contact is made to finalize it. On the impossibility of contacting them by phone, WhatsApp messages are sent offering the self-completion link. This application is also used to make calls, send messages with scheduling data and information about the research’s social media (Figure 1). 

### Field work team

Telephone interviews with the women were conducted by an all-female team of undergraduate students or health professionals. Interview with the men were conducted by interviewers of both sexes, from various fields, with at least a high school degree. All interviewers receive training to use the REDCap platform, are instructed on how to approach and conduct the interviews, and how to approach situations that may cause discomfort, such as reports of emotional stress and, for men, questions that address behavior and attitudes toward their partner. 

### First follow-up questionnaire with the new mothers 

The questionnaire is divided into eight blocks: (1) maternal morbidity after maternity discharge; (2) use of outpatient health services; (3) baby’s health; (4) satisfaction with abortion care; (5) post-traumatic stress; (6) mother-infant bond; (7) postpartum depression; and (8) anxiety.

Block 1 addresses the women’s health status after maternity discharge, investigating the presence/persistence of COVID-19 symptoms (extreme tiredness, shortness of breath, loss of smell and taste, limitation of activities, among others); readmission after maternity discharge (reason, location, and duration); and severe maternal morbidity (ICU admission, use of mechanical ventilation, blood transfusion, and hysterectomy). Block 2 consists of questions about postpartum consultations attended between 7 and 10 days after discharge, including advice on breastfeeding and contraception, contraceptive method use, and which method. Block 3 brings questions about the baby’s health, including hospitalization after maternity discharge (reason, location and duration), attendance of the childcare visit in the first week of life, exclusive and complementary breastfeeding, difficulty breastfeeding, BCG and hepatitis B vaccination, and neonatal heel prick and hearing screening. Block 4, applied only to those who had an abortion, consist of the instrument QualiAborto-Pt ^21^, which assesses the quality of care received in the maternity hospital from the women’s perspective. QualiAborto-Pt has 17 items, with five factors: admission, orientation, supplies/physical environment, technical quality and continuity of care. 

Blocks 5, 6, 7 and 8 specifically address postpartum mental health, and are applied to all women, except for block 6, which evaluates the mother-infant bond. 

Block 5 assesses childbirth-related post-traumatic stress disorder symptoms by means of the *City Birth Trauma Scale*, a 22-item instrument that measures the dimensions of intrusion, avoidance and mood alteration, developed by Ayers et al. ^22^ and validated in Brazil by Donadon et al. ^23^. 

Block 6 consists of the *Postpartum Bonding Questionnaire* (PBQ) ^24^, which aims to measure the quality of the postpartum mother-baby bonding. It consists of 25 items, with four factors: bonding problems (F1), rejection and pathological anger (F2), anxiety (F3), and risk of abuse (F4). The PBQ version that will be used in the *Birth in Brazil II* was developed and validated by Baldisserotto et al. ^25^.

Block 7 applies the *Edinburgh Postnatal Depression Scale* (EPDS) ^26^, a 10-item instrument that assess perinatal depressive symptomatology. The scale was validated for use by telephone interview in Brazil ^27^, and a cutoff point ≥ 10 indicates possible postpartum depression. 

Block 8 assesses anxiety symptoms using the *Generalized Anxiety Disorder scale* (GAD 7), translated into Brazilian Portuguese and available for free use at https://www.ph.qscreeners.com/sites/g/files/g10049256/f/201412/PHQ9_Portuguese%20for%20Portugal.pdf. It consists of seven items that measure the severity of anxiety disorder, and a cutoff point ≥ 10 suggests the presence of moderate/severe anxiety symptoms ^28^. 

### Second follow-up questionnaire with the new mothers 

The questionnaire has five thematic blocks: (1) symptoms of long COVID-19; (2) breastfeeding at four months; (3) abuse during parturition/abortion care; (4) satisfaction with care received at the maternity hospital; and (5) everyday discrimination.

Block 1 is applied only to women who have reported COVID-19 during pregnancy and comprises questions about persistence of symptoms, need for medical assistance, and severity and limitations due to the disease. This block contains the same questions as the first follow-up. 

Block 2 includes the breastfeeding questions from the first follow-up and is answered only by women whose babies are alive at the time of the interview. They answer four questions that assess the prevalence of exclusive and complementary breastfeeding.

Block 3 consists of 40 questions about abuse during parturition/abortion care based on the questionnaire elaborated by Bohren et al. ^29^. These questions assess physical violence, verbal violence, neglect, inappropriate vaginal touching, and stigma and discrimination. 

Block 4 refers to the women’s satisfaction with the care received during hospitalization, assessed by the *Satisfaction with Hospital Parturition Care Scale* ^30^. This instrument consists of 13 items and is applied only to postpartum women. 

In block 5, everyday discrimination is assessed using an adapted *Everyday Discrimination Scale* questionnaire, an 11-question instrument, 10 of which are about experiencing different discriminatory situations and one about the perceived reasons for the discrimination ^31^.

### Questionnaire for the babies’ fathers 

The questionnaire includes the following sets of variables: sociodemographic (place of residence, age, education, race/ethnicity, marital status, paid work, occupation); habits (smoking, alcohol consumption); general health (self-assessment of health, emotional problems, and current treatment); participation in pregnancy planning, prenatal care, and maternity care; relationship with the partner, measured by the *Paternal Adjustment and Paternal Attitudes Questionnaire* (PAPA); anxiety, measured by the *State-Trait Anxiety Inventory* (STAI); and depressive symptoms, measured by the *Edinburgh Postnatal Depression Scale* (EPDS).

PAPA is a multidimensional instrument on postpartum paternal adjustment and attitudes. It comprises 30 items divided into three subscales that assess the father’s relationship with the partner (sexual and conjugal) and their paternal attitudes. It is adapted from the *Maternal Adjustment and Maternal Attitudes Questionnaire* (MAMA) ^32^. 

STAI was developed by Spielberger et al. ^33^. In its original version, it consists of 20 questions divided into two scales: state-anxiety (STAI-S) and trait-anxiety (STAI-T). This study uses the short version validated in Brazil, consisting of six questions (STAI-S) ^34^.

Validated in Brazil, EPDS is often used in telephone interviews with postpartum women to measure depression ^27^. However, systematic review of studies on postpartum paternal depression that used the EPDS shows that the scale has acceptable accuracy for detecting symptoms of depression ^35^. 

### Strategies to reduce the non-response rate 

We adopted some strategies to avoid follow-up losses, reduce refusals, and increase the response rate. Besides the different forms of contact, the official website and the project’s social media (Instagram and Facebook) periodically publish posts about the importance of the survey, reminders about the telephone contacts, and information on how to identify possible fraud. The e-mail addresses of the social networks are printed on the refrigerator magnet given to the new mothers at the end of the hospital interview. 

As the father can only be contacted by the phone number provided by the woman, we developed promotional materials in the form of cards sent by WhatsApp, inviting them to participate and indicating the research’s social media where they can obtain information about the study and topics related to paternal health.

Since increasing the number of contacts reduces the non-response rate, further efforts are undertaken with those who did not answer initial contact attempts. Thus, new contact attempts are made 15 days after the scheduled interview date. 

Follow-up loss occurs when contact with the woman or partner is not possible by any means. When they clearly express that they do not wish to participate in the research, this is considered a refusal, and no further contact attempt is made. 

### Quality control of interviews 

Several strategies are used for quality control: (1) standardized training of the team of interviewers by the study coordination, with a description of the study and its objectives, logistics, reading of the questionnaires and instructions, and dynamics showing how the questionnaires should be applied; (2) questionnaire entered into the REDCap platform with critical data entry, avoiding implausible answers; (3) follow-up of field work and review of the questionnaires to identify incompleteness. For each incomplete questionnaire identified, the interviewer in charge must contact the respective participant to complete it; (4) inclusion of some questions from the hospital interview in the father’s questionnaires and in the mother’s follow-up, allowing to evaluate the agreement between the information, including the completion link, aiming at identifying possible frauds by the interviewer. In case of suspected fraud, new contact is made by the research coordination to confirm the data; (5) re-interview with inquiry of some questions on a random sample to confirm that the interview was conducted; and (6) calculation of interview time by recording the start and end time. This strategy serves to identify possible fraud in telephone interviews, but does not apply to self-completed questionnaires, which may take several hours or days to complete, depending on the participant’s availability. 

### Data analysis

Comparing the baseline data from *Birth in Brazil II* (maternity interview) with the follow-up interviews and the interviews with fathers will allow longitudinal and cross-sectional analyses. Descriptive analysis will be performed by calculating means and standard deviation for quantitative variables and frequency and percentage for categorical variables. Risk and prevalence measures and their confidence intervals will be estimated using univariate, stratified, and multiple model analysis. Models tested with different dependent and independent variables will be used to answer the various research questions. Various statistical analyses can be performed, either by means of generalized linear models or propensity scores. All models will be adjusted for potential confounders. Directed acyclic graphs (DAG) will be developed from a priori knowledge to investigate causality relations. Correlations between mental health variables (anxiety, depression, PTSD), possible mediating variables and the outcomes of interest will be estimated by Structural Equation Models (SEM). The analyses will also consider separately the telephone and self-completion link interviews, identifying possible biases associated with the medium. Statistical analyses will be performed using SPSS 26 (https://www.ibm.com/), R3.5.1 (http://www.r-project.org) and Mplus 8 (https://www.statmodel.com/).

*Birth in Brazil II* uses a complex sample and all analyses must consider the sample weights of the participants at each research stage. To correct non-response, we can: (1) probabilistically impute data from non-respondents; (2) model the response probability as a function of the variables obtained at baseline and use it to derive weight adjustments.

### Ethical aspects

The *Birth in Brazil II* survey was approved by the Brazilian National Ethics Research Committee (CONEP; opinion n. 3,909,299 on March 11, 2020). Survey with the babies’ fathers was submitted to the Research Ethics Committee Sergio Arouca National School of Public Health, Oswaldo Cruz Foundation (ENSP/Fiocruz) and approved on November 30, 2021 (opinion n. 5,136,883). Informed Consent Form (ICF) is applied to the postpartum women at the maternity ward, when authorization is also requested for contact 2 and 4 months after delivery. As for the partners, the ICF is read to them during the telephone interview and sent in PDF format by WhatsApp. Although the research involves some degree of risk, such as data leakage, embarrassment, and emotional triggering by the content of the questions, we established strategies to minimize these issues. To prevent data leakage, we will ensure confidentiality by using numerical codes to identify the interviewees. In case of embarrassment or emotional triggering, the participants may choose not to answer those questions considered more sensitive, without this causing any prejudice regarding participation in the research. If any emotional problem (anxiety, depression, PTSD) is identified, the data collection system reports the score obtained after application of the scales and, if the participant agrees, they will receive a list of places where they can seek perinatal mental health care in each participating city, as well as websites and help lines to contact, including for immediate support at the Brazilian Center for Valuing Life (CVV phone 188), available 24 hours a day, seven days a week. Once notified by the interviewers, the research coordination contacts the interviewee to provide guidance, and requests specialized emergency care.

## Discussion

In recent decades, Brazil has seen remarkable advances in maternal and childcare with improved health indicators, such as access to prenatal care, breastfeeding support, vaccination coverage, and reduction of mortality during the first year of life. But there is room for further advances in maternal and infant outcomes, encouraging attendance at postnatal consultations, still facing low coverage and several barriers five. Moreover, postnatal consultations should not focus solely on the biological issues of mother and baby, but consider other aspects of mothers’ and fathers’ lives, particularly the emotional aspects involved with the birth of a child. 

Globally, there has been a shift in the women’s and children’s health agenda from an exclusive focus on survival to the inclusion of prosperity and transformation factors. This change is a response to the third Sustainable Development Goal - ensure healthy lives and promote well-being for all at all ages (https://www.undp.org/sustainable-development-goals#good-health) - and the new Global Strategy for Women’s, Children’s and Adolescents’ Health ^36^. Similarly, the WHO guideline, *WHO Recommendation: Intrapartum Care for a Positive Childbirth Experience* ^37^ advocates not only technical recommendations to prevent maternal and newborn morbidity and mortality, but also encompasses a person-centered philosophy that includes optimizing women’s health and well-being. In this new conception, surviving parturition/birth is not enough. Women must enjoy a full social, physical, and mental well-being so that she and her baby can reach their full potential in life. 

Parenthood is a major life event that causes men and women to adapt to a wide range of biological, psychological, and sociocultural changes. It is a transformative experience that impacts the mental health of both, and increases the prevalence of depression, anxiety, and other mental disorders. Studies show that the quality of the marital relationship is negatively associated with depressive symptoms in both men and women, and positively associated with men’s involvement with pregnancy and father-infant interaction during postpartum ^38^ ^,^ ^39^. Countries such as the United Kingdom ^40^ and Australia ^41^ have already routinely adopted primary care as a priority site for investigating, treating, and following-up on perinatal depression according to well-established protocols. 

This study will estimate the prevalence of several indicators of mother and child health after birth, which will allow comparisons with the *Birth in Brazil I* survey. Moreover, it includes new topics such as paternal health, abuse in delivery care, and other characteristics that configure women’s vulnerability, enabling more detailed analyses and the correlation between indicators. The research also includes women who experienced abortion, allowing us to estimate its prevalence nationwide, something unprecedented in Brazil to date. Further, we intend to evaluate the correlations between various biological, behavioral, and psychological factors and maternal, paternal, and infant well-being from prenatal to postpartum data. Given the wide range of information collected, this study will allow us to draw a comprehensive profile of the health of new mothers and fathers. Our results may contribute to improve clinical practice and prevention efforts in public health.

Despite the methodological rigor adopted, some possible limitations of the study are those inherent to research conducted by telephone or online, such as answered calls, incorrect or nonexistent phone numbers, refusal to provide information over the phone or to access links sent by messaging apps. But as highlighted earlier, several strategies will be adopted to reduce losses. Another aspect is the exclusion of maternity hospitals with less than 100 deliveries/year. However, these institutions represent only 2% of births in the country, with a very small probability of introducing bias into the estimates. 

Some strengths should be highlighted. First, this is a unique study in Brazil. For the first time a large prospective cohort study will be conducted with national representation and a multifactorial approach to perinatal well-being, combined with measurements during delivery and postpartum. Second, the large study sample (22,050 postpartum women, their children, and partners) will allow us to observe possible correlations between psychological, physiological, and obstetric determinants. Finally, the sample size will allow us to validate various instruments and use them to answer the research questions.

## Conclusion

This study will provide evidence on postpartum maternal and paternal mental disorders, on the correlations between perinatal mental health, sociodemographic and obstetric factors and delivery/postpartum care, thus contributing to a better understanding of their occurrence and determinants in Brazil.
